# Bridging the gaps to overcome major hurdles in the development of next-generation tuberculosis vaccines

**DOI:** 10.3389/fimmu.2023.1193058

**Published:** 2023-08-11

**Authors:** Hongmin Kim, Han-Gyu Choi, Sung Jae Shin

**Affiliations:** ^1^ Department of Microbiology, Institute for Immunology and Immunological Diseases, Graduate School of Medical Science, Brain Korea 21 Project, Yonsei University College of Medicine, Seoul, Republic of Korea; ^2^ Department of Microbiology and Medical Science, College of Medicine, Chungnam National University, Daejeon, Republic of Korea

**Keywords:** *Mycobacterium tuberculosis*, next-generation TB vaccines, immune correlates, immunogenicity, biomarkers

## Abstract

Although tuberculosis (TB) remains one of the leading causes of death from an infectious disease worldwide, the development of vaccines more effective than bacille Calmette-Guérin (BCG), the only licensed TB vaccine, has progressed slowly even in the context of the tremendous global impact of TB. Most vaccine candidates have been developed to strongly induce interferon-γ (IFN-γ)-producing T-helper type 1 (Th1) cell responses; however, accumulating evidence has suggested that other immune factors are required for optimal protection against *Mycobacterium tuberculosis* (Mtb) infection. In this review, we briefly describe the five hurdles that must be overcome to develop more effective TB vaccines, including those with various purposes and tested in recent promising clinical trials. In addition, we discuss the current knowledge gaps between preclinical experiments and clinical studies regarding peripheral versus tissue-specific immune responses, different underlying conditions of individuals, and newly emerging immune correlates of protection. Moreover, we propose how recently discovered TB risk or susceptibility factors can be better utilized as novel biomarkers for the evaluation of vaccine-induced protection to suggest more practical ways to develop advanced TB vaccines. Vaccines are the most effective tools for reducing mortality and morbidity from infectious diseases, and more advanced technologies and a greater understanding of host-pathogen interactions will provide feasibility and rationale for novel vaccine design and development.

## 1 Introduction

Tuberculosis (TB), one of the deadliest infectious diseases, is caused by *Mycobacterium tuberculosis* (Mtb) and was responsible for approximately 1.6 million deaths in 2021 (World Health Organization. Global TB Report 2022). A single licensed TB vaccine called bacille Calmette-Guérin (BCG) has been employed for human use since 1921, and the degree of protection afforded by BCG vaccination varies in different regions of the world (1). Although the protective efficacy of BCG against severe TB forms such as TB meningitis and disseminated extrapulmonary TB before adolescence is well documented, worse protection with highly variable efficacies in individuals of all ages against pulmonary TB continues to be a serious concern (2, 3). Despite the global use of BCG for over 100 years, approximately a quarter of the world’s population is considered to have latent Mtb infection. Thus, the development of new TB vaccines that provide greater protection than the BCG vaccine, with the aim of preventing pulmonary TB, is critical for all age groups.

More than 20 TB vaccine candidates with various purposes have entered clinical trials, and 14 candidates are being actively evaluated. However, the unsatisfactory outcomes (for example, the MVA85A and AERAS-422 trials) (4–6) prompt us to try to further understand the complexity of the key protective immune response to Mtb infection and the way to develop vaccines that afford lifelong protection. These trials highlight our current knowledge gaps about protective correlates and controlling factors that can affect vaccine efficacies and outcomes. In this review, we discuss five points that should be considered in the individual stages of vaccine development, from the proposal of novel concepts for next-generation TB vaccines to considerations for practical development.

## 2 The first hurdle: purpose of vaccines

### 2.1 Prevention of infection

A vaccine developed for the prevention of infection (POI), given prior to Mtb exposure, should control the incipient infection stage. With much higher rates of infection than evident TB disease in endemic settings, POI trials are shorter and less costly than prevention of disease (POD) trials (7, 8). Therefore, the POI trial can be used as a viable opportunity to understand the mechanisms of vaccine efficacy in humans, providing a platform to select lead candidates for further testing. A major challenge is that there is no available standardized test to measure directly the acquisition, persistence, and clearance of asymptomatic Mtb infection. Currently, assessment of Mtb infection mainly relies on alterations in specific T-cell responses induced after Mtb infection. One of the commercial interferon (IFN)-γ release assay (IGRA), QuantiFERON-TB Gold In-Tube (QFT), measures immunological sensitization to Mtb as a biomarker for Mtb infection. Compared to persistent QFT negatives, recent negative-to-positive QFT tests are associated with higher rates of Mtb infection. Therefore, it may be ideal for conducting clinical trials of prevention of Mtb infection (POI) by novel vaccines using QFT transformation as an efficacy endpoint. A positivity cutoff IFN-γ value (0.35 IU/ml) for QFT conversion is recommended by manufacturers and CDC (9), but the immunological and analytical variability of QFT tests potentially confounds the interpretation of QFT conversion as a clinical trial endpoint (10, 11). Although the tuberculin skin test (TST) can be used as an alternative method for detecting Mtb infection, since specificity is reduced by BCG vaccination or nontuberculous mycobacteria (NTM) infection, novel diagnostic methods for successful clinical results must be developed.

### 2.2 Prevention of disease

A POD vaccine can be administered either pre- or post-exposure to protect against disease progression after actual Mtb infection. Knight et al. reported epidemiological modeling suggesting that adolescents or young adults are the most effective targets for POD vaccination (12). According to this model, due to children having lower rates of TB notifications, lower proportions of smear-positive pulmonary TB, and making a smaller contribution to TB transmission, a novel TB vaccine targeted at infants shows a reduced immediate impact compared to one targeted at adolescents/adults. Vaccines targeting infants prevent a relatively small number of active cases, resulting in fewer secondary cases being prevented. In contrast, vaccines targeting adolescents/adults directly affect the population with the greatest burden of active TB, such as 10-year-olds vaccinated in schools and those individuals reached in mass campaigns, which leads to a reduction in transmission. Although most vaccine candidates in clinical phases aim to prevent TB disease, POD trials require more time and higher costs than POI trials because of the much lower rate of TB disease than Mtb infection (8). Nevertheless, POD trials can directly reveal Mtb infection because the evaluation is performed by measuring clinical symptoms, chest X-ray, and direct Mtb culture from clinical samples. A recent POD trial with the candidate M72:AS01_E_ TB vaccine (phase 2b) was conducted in Kenya, South Africa, and Zambia. Efficacy analysis was conducted on a total of 3,283 subjects, and after a period of approximately 2.3 years, the incidence of pulmonary TB was significantly lower in the M72:AS01E group than in the placebo group (13). In this trial, M72:AS01E group showed 54% vaccine efficacy among persons already infected with Mtb, but due to the inclusion of predominantly BCG-vaccinated Mtb-infected adults, it was not possible to determine the extent to which infection-generated or childhood BCG vaccination-elicited responses influenced vaccine efficacy. Similarly, a 3-year extended follow-up study demonstrated 49.7% protection by M72:AS01_E_ among people already infected with Mtb (14), indicating that vaccine-induced protective immune responses were maintained for at least 3 years. With these promising findings, broader applications to diverse ethnic populations in different geographic settings will be required to conduct reliable clinical trials for POD.

### 2.3 Prevention of recurrence

Vaccines aimed at the prevention of recurrence (POR) are administered during antibiotic therapy to prevent the recurrence of TB. TB recurrence generally occurs in approximately 2 to 8% of TB patients even after treatment completion, and the recurrence rate depends on the absence or presence of cavities, bacterial burden, treatment frequency per week, type of antibiotics used and transmission rate. As most cases of recurrent TB disease develop within one year after treatment completion, the targeted populations of POR trials can usually be designated (8), but trial design is complicated due to the long-term treatment period and intervention timing. Multiple promising candidates currently under evaluation for POR include the H56:IC31 and ID93:GLA-SE subunit vaccine candidates, which were noted to prevent reactivation or restrict progression to severe disease in nonhuman primates (NHPs) (15, 16), and the recombinant BCG vaccine candidate VPM1002.

Due to the characteristics of TB, a large number of subjects for trials are needed because approximately 10% of infected individuals are at the onset of the disease. In addition, long-term monitoring is required because the timing of onset is different for each individual. These characteristics make the rapidly increasing economic problem more difficult as the number of clinical trial subjects and the test period increases. Therefore, to overcome these problems, it is important to recruit a reasonably sized experimental group and set endpoints according to the purpose of the experiment, and it is important to discover a correlate of protection (COP) that can predict vaccine efficacy, which will be addressed later in this review.

## 3 The second hurdle: a gap between experiments and the natural history of TB

Current concepts for the development of TB vaccines depend on experiments emphasizing T-helper type 1 (Th1)-biased immunity, based on early observations (17). For successful vaccine development, an appropriate vaccine model and translation to evaluate vaccine candidates is essential. Therefore, factors such as which animal model to select, which strain of Mtb to use for infection, and which dose to use for challenge are important.

### 3.1 Mtb infection dose

According to reports, the infection dose that causes TB disease is 1-200 colony-forming units (CFU). TB is transmitted via droplets containing Mtb generated through coughing or sneezing, and the number of droplets generated through a single cough is approximately 1-400 CFU (18, 19). According to another study of TB patients, the average number of aerosolized CFU generated by coughing for 5 minutes was 16 (20). When an individual is infected with Mtb, the actual infection dose may be much lower than the number of bacteria released by coughing because not all aerosols generated by the infected person are inhaled. In addition, it has been reported that symptomatic TB patients release Mtb aerosols not only by cough but also by exhalation, with an aerosol size of 0.5-5 μm, showing that actual infection can be achieved in the context of a sustained low-dose of bacteria (21, 22). However, animal models for vaccine research in the preclinical stage can be established through a single, sufficient infection dose and used to evaluate vaccine efficacy. It is unclear whether the reduction in the bacterial burden by vaccination in the context of single-dose infection is a good predictor of actual clinical vaccine performance. This single-dose challenge could overwhelm or bypass the relevant immunological cascade and mask the full potential of candidate vaccines (23). In a mouse model, ultra-low-dose aerosol infection with 1-3 CFU resulted in characteristics more similar characteristics to human TB, such as highly heterogeneous bacterial burdens and well-circumscribed granulomas, than conventional-dose infection with 50-100 CFU (24). Recently, Dijkman et al. tested the efficacy of pulmonary BCG vaccination in a rhesus macaque model with a 1 CFU Mtb infection every day for 11 days and noted the importance of Th17 cells and IL-10 (25). This study does not represent all situations in which infection occurs, but it does provide a model that accounts for specific persistent and practical infection situations, such as household contacts. These studies suggest that it is necessary to reconsider the infection dose used in preclinical vaccine studies.

### 3.2 Experimental models

Most individuals who become infected can remain asymptomatic for a long time. Although environmental, cultural, geographical, and contextual characteristics can affect whether infection occurs, TB susceptibility due to host genetic differences has been reported as one of the determinants of TB disease (26). In the case of TB studies, more than 60% of preclinical studies have used mouse models, susceptibility to Mtb differs depending on the mouse strain. The most widely used C57BL/6 mice or BALB/c mice have relatively low susceptibility, whereas DBA/2, CBA/J, I/St, C3H, and A/J strains have relatively high susceptibility (27). In addition, it has been reported that necrotic granulomas found in patients with active TB are not formed in BALB/c or C57BL/6 mice, whereas they are formed in the TB-susceptible mouse strains I/St and C3H (28, 29). Recently, it was confirmed that human-like necrotic granulomas were formed by Mtb infection in a humanized mouse model, and caseous necrotic granulomas showed an immune phenotype and spatial organization similar to those observed in TB patients (30). Arrey et al. presented the utility of this model for the evaluation of a preclinical model of anti-TB drugs in an *in vivo* environment. Zelmer et al. immunized different strains of inbred mice, such as A/J, DBA/2, C57BL/6, and 129S2, displaying different susceptibilities to Mtb with BCG (31). Smith et al. used a “collaborative cross” model system created by crossing inbred and outbred mice to understand the broader host genetic susceptibility spectrum and for use in vaccine efficacy testing (32). These models can confirm the genetic immunological correlation associated with TB vaccine efficacy and can simultaneously be used to identify potential improvements and key defense factors for the development of a robust TB vaccine.

To date, most studies with NHPs have been used to model only acute TB, which is much less prevalent than latent TB in humans (33). Rhesus macaques develop active TB in approximately 90% of the infected population, whereas cynomolgus macaques develop active TB in only 60% of the infected population. It has also been reported that BCG showed a higher protective efficacy in cynomolgus macaques than in rhesus macaques (34). Rhesus macaques and cynomolgus macaques can develop acute, chronic, or latent TB depending on the route of infection, dose, and Mtb strain used for inoculation.

Vaccine trials employing the NHP model are expensive, but they can serve as a checkpoint for clinical trials, resulting in significant cost savings. Areas-402 induced a strong T-cell response but did not protect rhesus macaques against infection with 200 CFU of Mtb Erdman (35). Conversely, a clinical study of MVA85A without efficacy evaluation was performed with the NHP model, and although it was expensive, the efficacy was not proven in the clinical stage (36, 37). These results suggest that validation of efficacy for TB vaccines via the NHP model to enter the clinical stage may accelerate TB vaccine development.

In an evaluation of vaccine efficacy in animal models, the bacterial burden between vaccinated and non-vaccinated groups is one of the key factors. Previous studies have demonstrated the presence of non-replicating bacterial populations in sputum samples obtained from TB patients (38, 39). These non-replicating subpopulations have been attributed to the existence of persister-like bacilli in a non-replicating state (39). Even in preclinical TB vaccine models, nonculturable or persistent mycobacterial subpopulations may arise due to immunological pressures resulting from the characteristics of the vaccine candidate or vaccine model, which can hinder the accurate evaluation of vaccine efficacy. Resuscitation-promoting factors (Rpfs) are bacterial proteins which are primarily identified by their ability to resuscitate nonreplicating cells *in vitro* and *in vivo* (40, 41). It has been reported that non-replicating bacteria in a patient’s sputum can be revived by Rpfs and the culture time can be shortened (42, 43). The application of Rpfs to bacterial culture in conventional media has the potential to reduce errors in vaccine efficacy evaluation that can be caused by nonculturable or persistent subpopulations.

### 3.3 Translation and interpretation: differential analysis of samples between humans and animals

NHPs show anatomical and physiological similarities with humans as well as a wide range of clinical symptoms of TB, including pulmonary and extrapulmonary signs and symptoms. The NHP model enables the analysis of infected tissue, which is difficult in clinical stages, and at the same time, the disease course can be monitored by measuring parameters on radiographic images and examining body fluid samples, which can also be performed in humans. In addition, the NHP model allows the use of computed tomography and positron emission tomography to observe the progression of Mtb infection to disease in an individual (44). In the clinical phase, blood samples are used to measure immunogenicity. Currently, the Ag-specific T-cell response, multifunctionality of the Ag-specific T-cell response, and Ag-specific IgG antibody titers are commonly evaluated to demonstrate immunogenicity after vaccination (**Table 1**). However, in the case of vaccine candidates, when the efficacy is evaluated through animal experiments, the analysis is not based on blood but rather on tissues, such as lung, spleen, and lymph nodes. Therefore, it is difficult to apply COPs from tissue-based analysis in preclinical studies to clinical studies. The NHP model enables analysis of indicators applicable to human clinical studies such as blood, urine, and PET-CT results, and analysis of indicators that can be measured only after sacrificing animals, which is possible only in preclinical models. Exploration and verification of significant indicators through this model can lead to an acceleration of vaccine development. Therefore, studies employing the NHP model before the clinical stage can provide meaning beyond simply being the gateway to the clinical stage.

**Table 1:** Common and specific immunogenicity assessments of TB vaccines in clinical trials.

Type of vaccine	Name of vaccine	Purpose	Phase	Immunogenicity assessment	Reference
**Live** **attenuated** **vaccine**	MTBVAC	POD	3	▪ Frequencies of MTBVAC-specific CD4^+^/CD8^+^ T cells producing one or more cytokines (IFN-γ, TNFα, IL-2, IL-17, or IL-22) ▪ IFN-γ response to stimulation with ESAT6 and CFP10 in whole blood ▪ IFN-γ ELISpot assay with PBMCs	(45, 46)
	VPM1002	POI, POD, POR	3	▪ Concentration of IFN-γ upon PPD stimulation in whole-blood samples determined by ELISA ▪ Proportions of distinct subsets of specific CD4^+^/CD8^+^ T cells produced one or more cytokines (IFN-γ, TNF-α, and/or IL-2) simultaneously in whole blood samples in response to PPD stimulation ▪ PPD- and Ag85B-specific antibodies (IgG, IgA, and IgM) in serum	(47–49)
	BCG revaccination	POI, POD	3	▪ Frequencies of BCG-specific CD4^+^/CD8^+^ T cells expressing at least two of three cytokines (IL-2, IFN-γ, and TNF-α) ▪ Change in the concentration of IFN-γ in blood samples ▪ CD4^+^ T-cell subsets expressing IL-17A, IL-17F, or IL-22 (Th17) - either in combination with IFN-γ or IL-10 ▪ Frequencies of NKT cells, γδ T cells, and CD56^hi/dim^ NK cells producing IFN-γ	(50–52)
**Adjuvanted** **protein** **subunit** **vaccine**	M72/AS01_E_	POD	3	▪ The titer of M72-specific IgG antibody in serum ▪ Frequencies of M72-specific CD4^+^/CD8^+^ T cells expressing one or more cytokines (IFN-γ and/or IL-2 and/or TNF-α and/or CD40L) simultaneously in PBMCs ▪ IFN-γ production by CD69^+^CD56^+^ NK cells after stimulation with M72 peptide pool in PBMCs	(53–56)
	GamTBvac	POD	3	▪ Frequencies of vaccine Ag-specific CD4^+^/CD8^+^ T cells expressing IFN-γ, TNF-α, and/or IL-2 in blood samples ▪ Change in the concentration of IFN-γ in blood samples ▪ The titer of IgG specific to the subunits of GamTBvac (fusion forms, DBD-Ag85A and DBD-ESAT6-CFP10, and subsets Ag85A, ESAT6, CFP10, and DBD)	(57, 58)
	ID93/GLA-SE (QTP-101)	POI, POD	2b	▪ Frequencies of cytokine-expressing CD4^+^ T cells specific to ID93, Rv1813, Rv2608, Rv3619, and Rv3620 ▪ IFN-γ and IL-10 cytokine-secreting cells in PBMCs in response to ID93 determined by ELISpot ▪ Frequencies of ID93 specific-CD4^+^/CD8^+^ T cells producing one or more cytokines (IFN-γ, TNF, and IL-2) in PBMCs ▪ Titer of total IgG specific to ID93 and each fusion-protein antigen component (Rv1813, Rv2608, Rv3619, and Rv3620) ▪ Titer of ID93-specific total IgG, IgG1, IgG2, IgG3, and IgG4	(59, 60)
	H56/IC31	POR	2b	▪ Frequencies of CD4^+^ T cells expressing IFN-γ, TNF-α, IL-2 and/or IL-17 after stimulation with H56-fusion protein or peptide pools of Ag85B, ESAT-6 or Rv2660c in whole blood samples ▪ Memory phenotypes of H56-specific cytokine-expressing CD4^+^ T cells (IFN-γ^+^, TNF-α^+^, and/or IL-2^+^) ▪ Titer of IgG specific to H56 in plasma samples determined by ELISA	(52, 61)
	AEC/BC02	POD	2a	▪ Evaluation of IFN-γ and antibody level in blood before and after immunization determined by intracellular cytokine staining ▪ Changes in the levels of Ag-specific total IgG antibodies and IgG subclasses (IgG1 and IgG2) ▪ Changes in the levels of Ag-specific IFN-γ levels ▪ The changes in the proportion of Ag-specific T cells in PBMCs ▪ Evaluation of ex vivo intracellular cytokine staining and ELISpot results in blood	(62)

POD, prevention of disease; POI, prevention of infection; POR, prevention of recurrence; ELISpot, enzyme-linked immunospot; PBMCs, peripheral blood mononuclear cells; PPD, purified protein derivative; ELISA, enzyme-linked immunosorbent assay; BCG, bacille Calmette-Guerin, * This paper focuses on subunit vaccines and live attenuated vaccines. Other TB vaccines in clinical studies, such as killed mycobacteria vaccines and viral vectored vaccines, are reviewed in detail in other articles (63, 64).

## 4 The third hurdle: antigen selection

### 4.1 Universal antigens

Mtb contains approximately 4,000 individual proteins, and most Ags included in current subunit vaccines have been adopted mainly based on their immunodominant properties for T-cell responses in preclinical and clinical settings. Currently, approximately 100 Ags in the preclinical stage (approximately 3% of all Mtb Ags) have been studied (**Table 2**). Most of the Ags for TB vaccine candidates are abundantly secreted and cell wall-associated proteins, including ESAT6, Ag85B, Ag85A, HSPX, and MPT64. In addition, cell wall-associated or virulence-associated Pro-Glu/Pro-Pro-Glu (PE/PPE) family proteins, a component of M72 subunit and ID93 subunit vaccine candidates, and heparin-binding hemagglutinin also produced promising vaccine-induced protection in mouse models (103, 104). Ags related to latency (DosR, resuscitation-promoting factor) and hypoxia-related proteins are being used for vaccine testing. Furthermore, hypothetical proteins are also used, for example, Rv1767, which is produced by the pathogen during the first week of infection of human cells. Aagaard et al. reported that the dimers EsxD-EsxC, EsxG-EsxH and EsxW-EsxV produced by the ESAT6 secretion system (ESX) were highly immunogenic. Integrating these in a fusion protein form called H65 resulted in a formulation that demonstrated protection efficacy equivalent to that of BCG without interfering with current ESAT6- and CFP10-based diagnostics (105). Liu et al. also purified 1,250 Mtb proteins with an *E. coli* expression system and evaluated cellular and humoral immune responses in human PBMCs and serum, respectively. They eventually identified four Ag candidates, Rv0232, Rv1031, Rv1198, and Rv2016, displaying high immunogenicity (106). Currently, only 11 Ags have been selected as a component, in the form of fusion proteins, in formulations eventually entered into clinical trials (**Table 3**).

**Table 2:** Mtb antigens identified from preclinical experiments as vaccine components.

Gene accession No.	Antigen name	Rationale	Vaccine type	Route	Booster	Immunological role	Reference
Rv0129c	Ag85C	-	Recombinant bacterial (*L. ivanovii*)	IN	-	IgA secretion; Th1/Th17, TNF-α^+^IL-17^+^ CD8^+^ T cells	(65)
Rv0159c	PE3	Elicit T-cell responses	Recombinant bacterial (*M. smegmatis*)	IP	-	IL-2/IFN-γ secretion (Th1 response)	(66)
Rv0160c	PE4	-	Recombinant bacterial (*M. smegmatis*)	IP	-	IL-2/TNF-α/IL-6 secretion	(67)
Rv0288	CFP7	Early-stage antigen	Fusion component (protein vaccine)	SC	-	IFN-γ/IL-17 secretion	(68)
Rv0288	CFP7	Early-stage antigen	Fusion component (protein vaccine)	SC	-	IFN-γ/IL-17 secretion	(69)
Rv0572c	DosR	Latency-associated hypothetical protein	Single protein	SC	-	IgG2a/IgG1 ratio, IFN-γ/TNF-α/IL-2 secretion (Th1 response)	(70)
Rv0577	TB27.3	Secreted by actively replicating bacteria	Fusion component (DNA vaccine)	ID	-	IgG2a/IgG1 ratio, IFN-γ/TNF-α secretion (Th1 response), IFN-γ^+^ T_EM_ and IL-2^+^ T_CM_ cells (memory T cells)	(71)
Rv0733	ADK	Screening based on cellular and humoral responses in active TB patients	Single protein	SC	-	IFN-γ^+^TNF-α^+^IL-2^+^ CD4^+^/CD8^+^ T cell cells	(72)
Rv0915c	PPE14 (Mtb41)	Screening of Mtb expression library with specific T-cell lin	Single antigen (DNA vaccine)	IM	-	IgG2a secretion, IFN-γ secretion (Th1 response)	(73)
Rv0916c	PE7 (Mtb10)	Screening of Mtb expression library with specific T-cell line	Single antigen (DNA vaccine)	IM	-	IgG2a secretion, IFN-γ secretion (Th1 response)	(73)
Rv1009	RpfB	Reactivation	Single antigen (DNA vaccine)	IV	-	IL-2/IFN-γ secretion (Th1 response)	(74)
Rv1009	RpfB	Reactivation	Fusion component (protein vaccine)	SC	-	IgG2a/IgG1 ratio, IFN-γ/TNF-α/IL-2 secretion (Th1 response), IL-2^+^ multifunctional (TNF-α or IFN-γ) CD4^+^/CD8^+^ T cells	(75)
Rv1009	RpfB	Reactivation	Fusion component (protein vaccine)	SC	-	IgG2a/IgG1 ratio, IFN-γ secretion (Th1 response), IFN-γ^+^ T_EM_ IL-2^+^ T_CM_ (memory T cells), multifunctional (IFN-γ/TNF-α/IL-2) CD4^+^/CD8^+^ T cells	(76)
Rv1009	RpfB	Reactivation	Single antigen (protein vaccine or DNA vaccine)	SC or IM	-	IFN-γ^+^TNF-α^+^IL-2^-^CD107^+^ CD4^+^/CD8^+^ T cells	(77)
Rv1039c	PPE15	Possible secreted antigen	Single antigen (ChAdOx1 viral vector)	IN or ID	- and +	IFN-γ^+^/TNF-α^+^/IL-17^+^ CD4^+^ T cells, CD45^-^CXCR3^hi^KLRG^lo^ CD4^+^/CD8^+^ T cells	(78)
Rv1174c	TB8.4	Extracellular proteins expressed by replicating bacilli	Fusion component (protein vaccine)	SC	BCG booster	IFN-γ^+^/IL-17^+^ CD4^+^ T cells	(69)
Rv1174c	TB8.4	Extracellular proteins expressed by replicating bacilli	Fusion component (protein vaccine)	SC	- and +	IFN-γ^+^ CD4^+^ T cells, IgG2a/IgG1 ratio	(79)
Rv1285	CysD	-	Fusion (CysVac2/Advax^CpG^)	IM or ID	-	Multifunctional (IFN-γ/TNF-α/IL-2) CD4^+^/CD8^+^ T cells, local accumulation of neutrophils (CD45^+^CD11b^+^Ly6G^+^) and CD64^+^ macrophages/monocytes (CD45^+^CD64^+^CD11b^+^Ly6G^−^)	(80)
Rv1419	Unknown	Secreted during proliferation	Single antigen (DNA vaccine-pVAX1 vector)	IM	therapeutic	IFN-γ^+^ CD4^+^ T cells	(81)
Rv1485	hemZ	IFN-γ release in PBMCs from hospitalized TB patients determined using IFN-γ ELISpot assays	Single protein	SC	-	IgG2a/IgG1 ratio, IL-2/TNF-α/IL-6/IFN-γ secretion	(82)
Rv1503c	Conserved protein (glycolipid synthesis)	Regulator PhoPR	Single protein (rBCG, live vaccine)	SC	-	IFN-γ secretion (Th1 response), Multifunctional (IFN-γ/TNF-α/IL-2) CD4^+^/CD8^+^ T cells	(83)
Rv1705c	PPE22	IFN-γ release in PBMCs from hospitalized TB patients determined using IFN-γ ELISpot assays	Single protein	SC	-	IgG2a/IgG1 ratio, IL-2/TNF-α/IL-6/IFN-γ secretion	(82)
Rv1733c	DosR	Latency	Single protein or peptide	SC	-	IFN-γ^+^ CD4^+^ T cells	(84)
Rv1738	Unknown	Hypoxic	Fusion component (live vaccine; yeast-based platform)	ID	- or therapeutic	IFN-γ^+^IL-17^+^ CD4^+^ T cells	(85)
Rv1767	Hypothetical protein	Might be relevant for intracellular survival	Single protein (rBCG, live vaccine)	ID	-	IFN-γ^+^/IL-17^+^ CD4^+^ T cells	(86)
Rv1793	EsxN	Virulence factor	Fusion component (protein vaccine)	SC	-	TNF-α^+^/IL-17^+^ secretion ratio	(87)
Rv1876	bfrA	Screening from a fraction that strongly induced the activation of immune cells	Single protein	SC	BCG booster	Multifunctional (IFN-γ/TNF-α/IL-2) CD4^+^ T cells	(88)
Rv1886c*	Ag85B	Extracellular proteins expressed by replicating bacilli	Fusion component (protein vaccine)	SC	-	IFN-γ^+^/IL-17^+^ CD4^+^ T cells	(69)
Rv1886c*	Ag85B	Extracellular proteins expressed by replicating bacilli	Fusion (CysVac2/Advax^CpG^)	IM or ID	-	Multifunctional (IFN-γ/TNF-α/IL-2) CD4^+^/CD8^+^ T cells, local accumulation of neutrophils (CD45^+^CD11b^+^Ly6G^+^) and CD64^+^ macrophages/monocytes (CD45^+^CD64^+^CD11b^+^Ly6G^−^)	(80)
Rv0228	TB10.4	BCG antigens	Fusion component (protein vaccine)	SC	BCG booster	IFN-γ^+^ CD4^+^/CD8^+^ T cells	(89)
Rv0228	TB10.4	Antimycobacterial immune responses in BCG-immunized humans	Fusion component (viral vector)	IN	BCG-virus vaccine	Multifunctional (IFN-γ/TNF-α, IFN-γ/IL-2) CD44^+^CD62L^-^CD4^+^/CD8^+^ T cells, IFN-γ^+^ CD4^+^ T cells	(90, 91)
Rv2005c	Universal stress protein family protein	Latency	Fusion component (protein vaccine)	SC	Immunotherapy	Muti-functional (IFN-γ/IL-2/TNF-α) CD44^+^CD4^+^ T cells, IFN-γ/IL-2/IL-17 secretion	(92)
Rv2031c	HspX	BCG antigens	Fusion component (protein vaccine)	SC	BCG protein	IFN-γ^+^ CD4^+^/CD8^+^ T cells	(89)
Rv2031c	HspX	Immunoadjuvant potential	Fusion component (viral vector)	IN	BCG-virus vaccine	Multifunctional (IFN-γ/TNF-α, IFN-γ/IL-2) CD44^+^CD62L^-^CD4^+^/CD8^+^ T cells, IFN-γ^+^ CD4^+^ T cells	(90, 91)
Rv2031c	HspX	Expressed at the proliferating and dormant stages	Fusion component (protein vaccine)	SC	–	IgG2a/IgG1 ratio, IFN-γ/TNF-α/IL-2 secretion (Th1 response)	(93)
Rv2031c	HspX	Dormancy-related antigen	Fusion component (protein vaccine)	SC	–	IFN-γ^+^ CD4^+^ T cells	(94)
Rv2031c	HspX	Dormancy-related antigen	Recombinant bacterial (BCG expressed) fusion protein	Tail	–	IFN-γ^+^ CD4^+^ T_EM_ and IL-2^+^ CD8^+^ T_CM_ cells	(95)
Rv2031c	HspX	Dormancy-related antigen	Recombinant bacterial (BCG expressed) fusion protein	SC	–	IFN-γ^+^/IL-17^+^ CD4^+^ T cell, IFN-γ^+^IL2^+^ CD4^+^ T cells	(96)
Rv2032	acg	Hypoxic	Recombinant bacterial (*S. cerevisiae* yeast expressed) fusion protein	SC and ID	BCG-yeast	IFN-γ^+^IL-17^+^ CD4^+^ T cells	(85)
Rv2034	ArsR repressor protein	Potential function in Mtb survive in the host	Fusion component +H1 (protein vaccine)	SC		Multifunctional (IFN-γ/TNF-α) CD4^+^ T cells	(97)
Rv2073c	Probable short chain dehydrogenase	Hypoxic	Fusion component (DNA vaccine)	IM	–	IgG2a/IgG1 ratio, IFN-γ/TNF-α secretion (Th1 response), IFN-γ^+^ T_EM_ IL-2^+^ T_CM_ cells (memory T cells)	(71)
Rv2299c	htpG	Screening from a fraction that strongly induced the activation of immune cells	Fusion component (protein vaccine)	SC	+	Multifunctional (IFN-γ/IL-2/TNF-α) CD4^+^ T cells	(98)
Rv2608	PPE42	Virulence factor	Fusion component (protein vaccine)		-	TNF-α^+^/IL-17^+^ secretion ratio	(87)
Rv2628	unknown	Latency	Fusion component (protein vaccine)		-	TNF-α^+^/IL-17^+^ secretion ratio	(87)
Rv2875	Mpt70	Hypoxic	Fusion component (DNA vaccine)	IM	–	IgG2a/IgG1 ratio, IFN-γ/TNF-α secretion (Th1 response), IFN-γ^+^ T_EM_ IL-2^+^ T_CM_ cells (memory T cells)	(71)
Rv3019c	esxR	Immunodominant and immunogenic *in vivo*-expressed TB proteins in Mtb-exposed individuals	Fusion component (protein vaccine)	SC	-	Multifunctional (IFN-γ/IL-2/TNF-α) CD4^+^ T cells, KLRG^-^ CD4^+^ T cells	(99)
Rv3020c	esxS	Immunodominant and immunogenic *in vivo*-expressed TB proteins in Mtb-exposed individuals	Fusion component (protein vaccine)	SC	-	Multifunctional (IFN-γ/IL-2/TNF-α) CD4^+^ T cells, KLRG^-^CD4^+^ T cells	(99)
Rv3044	fecB	Hypoxic	Fusion component (DNA vaccine)	ID	-	IgG2a/IgG1 ratio, IFN-γ/TNF-α secretion (Th1 response), IFN-γ^+^ T_EM_ IL-2^+^ T_CM_ cells (memory T cells)	(71)
Rv3131	DosR	Hypoxic	Single protein	SC	-	Multifunctional (IFN-γ/IL-2/TNF-α) CD44^+^CD62L^-^CD4^+^ T cells, IFN-γ secretion	(100)
Rv3130	tgs1	Hypoxic	Recombinant bacterial (*S. cerevisiae* yeast expressed) fusion protein	SC and ID	BCG-yeast	IFN-γ^+^IL-17^+^ CD4^+^ T cells	(85)
Rv3329	Unknown	Immunogenic proteins	Single protein	SC	-	IFN-γ/TNF-α/IL-2/IL-12/IL17 secretion	(101)
Rv3407	vapB47	Latency	Fusion component (protein vaccine)	SC	-	IgG2a/IgG1 ratio, IFN-γ secretion (Th1 response), IFN-γ^+^ T_EM_ IL-2^+^ T_CM_ cells (memory T cells), Multifunctional (IFN-γ/TNF-α/IL-2) CD4^+^/CD8^+^ T cells	(76)
Rv3432c	gadB	Immunogenic proteins	Single protein	SC	-	IFN-γ/TNF-α/IL-2/IL-12/IL17 secretion	(101)
Rv3615c	EspC	Non-BCG antigens	Fusion component (protein vaccine)	SC	BCG booster	IFN-γ^+^ CD4^+^/CD8^+^ T cells	(89)
Rv3616c	EspA	Non-BCG antigens	Fusion component (protein vaccine)	SC	BCG booster	IFN-γ^+^ CD4^+^/CD8^+^ T cells	(89)
Rv3803c	MPT51	Immunodominant antigens	Recombinant bacterial (BCG expressed) fusion protein	SC	–	IFN-γ^+^/IL-17^+^ CD4^+^ T cells, IFN-γ^+^IL2^+^ CD4^+^ T cells	(96)
Rv3804c*	Ag85A	–	Recombinant bacterial (BCG expressed) fusion protein	Tail	–	IFN-γ^+^ CD4^+^ T_EM_ and IL-2^+^ CD8^+^ T_CM_ cells	(95)
Rv3841	bfrB	Hypoxic	Recombinant bacterial (*S. cerevisiae* yeast expressed) fusion protein	SC and ID	BCG-yeast	IFN-γ^+^IL-17^+^ CD4^+^ T cells	(85)
Rv3873	PPE68	Immunodominant antigens	Fusion component (protein vaccine)	SC and ID	BCG booster	Multifunctional (IFN-γ/IL-2/TNF-α/IL-17) CD4^+^ T cells	(102)
Rv3874*	CFP10, esxB	Non-BCG antigens	Fusion component (protein vaccine)	SC	BCG booster	IFN-γ^+^ CD4^+^/CD8^+^ T cells	(89)
Rv3875*	ESAT6, esxA	Non-BCG antigens	Fusion component (protein vaccine)	SC	BCG booster	IFN-γ^+^ CD4^+^/CD8^+^ T cells	(89)
Rv3875*	ESAT6, esxA	Virulence factor	Fusion component (protein vaccine)	SC	–	IFN-γ^+^ CD4^+^ T cells	(94)

PBMCs, peripheral blood mononuclear cells; BCG, bacille Calmette–Guérin; rBCG, recombinant BCG; T_EM_ cell, effector memory T cell; T_CM_ cell, central memory T cell, ELISpot, enzyme-linked immunospot; IN, intranasal; IP, intraperitoneal; ID, intradermal; IM, intramuscular; IV, intravenous; SC, subcutaneous; ^*^Antigens in a clinical trial for a TB vaccine.

**Table 3:** Composition and selection of subunit vaccines in the clinical stage.

	Composition	Ag selection	Reference
**GamTBvac**	▪ Ag85A and ESAT6-CFP10 fusion protein/DEAE-dextran-CpG adjuvant	▪ Induces strong IFN-γ production and T-cell proliferation (Ag85A) ▪ The fusion of Mtb early-secreted Ag (ESAT6, CFP10)	(50, 107, 108)
**M72/AS01_E_**	▪ Mtb32A-Mtb39A fusion protein/AS01_E_ adjuvant	▪ Recognized by PBMCs of healthy, disease-free, PPD-positive donors (Mtb32A, Mtb39A) ▪ Induces strong T-cell proliferation and IFN-γ production (Mtb32A, Mtb39A)	(53–56, 109, 110)
**H56/IC31**	▪ Ag85B-ESAT6-Rv2660c fusion protein/IC31 adjuvant	▪ Early-secreted Ags (Ag85B, ESAT6) ▪ Sustained secretion levels in the early and late stages of infection (Rv2660c)	(61, 111, 112)
**ID93/GLA-SE**	▪ Rv1813-Rv2608-Rv3619-Rv3620 fusion protein/GLA-SE adjuvant	▪ A hypothetical protein enriched under hypoxic growth (Rv1813) ▪ A probable outer membrane-associated Pro-Pro-Glu (PPE) motif-containing protein (Rv2608) ▪ Secreted proteins belonging to the ESAT6 family (Rv3619, Rv3620)	(59, 60, 113)
**AEC/BC02**	▪ ESAT6-CFP10 fusion protein and Ag85B/BC02 adjuvant	▪ Strongly recognized by T cells in the early phase of infection (CFP10, ESAT6, Ag85B)	(114, 115)

### 4.2 Rational antigen selection

The challenge of Ag screening is complicated by the roles of Ags in multiple stages of Mtb infection, particularly chronic and latent infection stages. During Mtb infection in a mouse model, ESAT6 is consistently expressed, but Ag85B is mainly expressed at an early stage when Mtb is actively replicating (116). Mtb infection induced the accumulation of ESAT6-specific CD4^+^ T cells in the mouse lung parenchyma, but the T cells became functionally exhausted due to chronic stimulation of Ag. Whereas, Ag85B-specific CD4^+^ T cells maintain memory cell features during infection but contract in numbers by reduced Ag expression during persistent infection (116). These results have important implications for the rational design of TB vaccines tailored to optimize the protection conferred by specific CD4^+^ T cells that recognize Ag expressed at distinct stages of Mtb infection.

Although immunodominant Ags are generally accepted to induce superior vaccine efficacy, some studies suggest that Mtb can modulate host immunity through immunodominant Ags. T-cell epitopes among well-known immunodominant Mtb Ags are highly conserved, suggesting the possibility that being recognized by the host through immunodominant Ags may be beneficial for Mtb (117, 118). In addition, T-cell responses to some immunodominant Mtb Ags have been found to be notably greater in active TB patients than in individuals latently infected with Mtb (119, 120), indicating that enhanced T-cell responses may be associated with deteriorated lung inflammation, resulting in subsequent transmission. Therefore, to confirm this possibility, Orr et al. confirmed the efficacy of immune subdominant Ags as a TB vaccine candidate in a mouse model, but little correlation has been found between vaccine efficacy and the immunodominance of Ags during Mtb infection (121).

Furthermore, it is also important to characterize the vaccine potential of Ags likely to be associated with reactivation from latent Mtb infection. A well-characterized bacterial regulon can induce the dormant state of Mtb that is controlled by DosR–DosS, which is induced by immunological pressure of the host such as local hypoxia, nitric oxide, and carbon monoxide (122). These host immune responses can be induced by the vaccine with immunodominant Ag in an active state of Mtb, indicating the potential for immune evasion of Mtb against immune responses produced by vaccines targeting immunodominant Ags in an activated state. Therefore, it is also important to characterize the vaccine potential of Ags likely to be associated with reactivation of latent Mtb infection. Hence, developing novel vaccines that encode genes expressed during the reactivation of a dormant state, such as *rpf*, would be a strategic approach. *In vitro* and *in vivo* transcriptional profiling studies have shown that five *rpf* of Mtb are expressed at varying levels in a growth stage-dependent manner (123, 124). RpfB, one of the five Rpfs produced by Mtb, plays an important role in the resuscitation and growth in a dormant state. In addition, delayed reactivation induced by aminoguanidine in chronic TB was observed in mice infected with a strain lacking *rpfB* (125), and significantly higher T cell responses to recombinant RpfB and RpfE were detected in LTBI than in active TB patients (126), indicating that Rpfs are involved in the reactivation process *in vivo*. Moreover, RpfB has been studied as a promising candidate for DNA vaccines, shown to induce a modest but significant cellular immune response against TB with higher levels of IL-2 and IFN-γ (74). In addition, the RpfB domain can induce a humoral response, and monoclonal antibodies against Rpfs could inhibit TB relapse (127).

### 4.3 Strategy for fusion proteins

Vaccine strategies using fusion proteins can be designed to include multiple Ags, so they can induce a broader immune response than single Ag vaccines. In addition, this strategy has the advantage of inducing an effective immune response by fusing a protein with low immunogenicity with a protein or peptide with high immunogenicity. The M72 vaccine candidate was formed through a fusion of the Mtb32A and Mtb39A proteins, selected for their ability to provoke T-cell responses in TST-positive healthy adults. On the other hand, multistage subunit vaccines, such as H56 (which contains the latency-associated Ag Rv2660c fused with Ag85B and ESAT6), as well as LT70 and ID93, also incorporate multiple Mtb Ags differentially involved in bacterial growth, virulence, and metabolism (111, 128, 129). A new TB vaccine candidate, H107, that integrates eight individually protective Ags (PPE68, ESAT6, EspI, EspC, EspA, MPT64, MPT70, and MPT83) is highly immunogenic in both mice and humans (102). This fusion protein is composed of 4 ESAT6 molecules in the middle, which led to a significant increase in ESAT-6-specific immunogenicity. H107 with the BCG vaccine could increase Ag coverage to induce robust protective immune responses in a diverse human population by including as many protective/recognizable Ags as possible. While traditional vaccines containing BCG-shared Ags show *in vivo* cross-reactivity to BCG, H107 demonstrates no cross-reactivity and does not impede BCG colonization. Instead, co-administering H107 with BCG results in enhanced adaptive responses against both H107 and BCG (102).

## 5 The fourth hurdle: immune correlates and protection biomarkers

Unveiling reliable predictive correlates to the immunogenicity and efficacy of TB vaccines allows estimation of vaccine efficacy well in advance of the time required to confirm vaccine efficacy against Mtb infection in the clinical stage. In addition, after the commercialization of a vaccine, successful vaccination can be tracked through reliable COP measurement of vaccinated individuals, and as a result, herd immunity through vaccination can be effectively achieved. Therefore, attempts have been made to identify reliable COPs of TB vaccines, but it is still unclear. Currently, in most vaccine studies in clinical phases, immunogenicity or vaccine-induced protection-related biomarkers are limited to immunological markers, especially IFN-γ-producing T cell responses, polyfunctional T-cell responses, or antibody titers in response to Mtb Ag. Recently, the possibility of developing protective immunity and vaccines for donor unrestricted T cells (DURTs), Th17 cells, antibodies, B cells, and innate immunity beyond Th1 immunity has been reconsidered for TB vaccines (130). Moreover, the association between TB progression and type I IFNs in active TB disease has been reported, but clinical studies on the efficacy and markers of TB vaccines are still lacking. These results raise questions about the sufficiency of T-cell responses induced by vaccination for protection and force us to explore additional biomarkers of vaccine efficacy.

### 5.1 Correlates of protection in innate immune response to the TB vaccine

**Figure 1:**
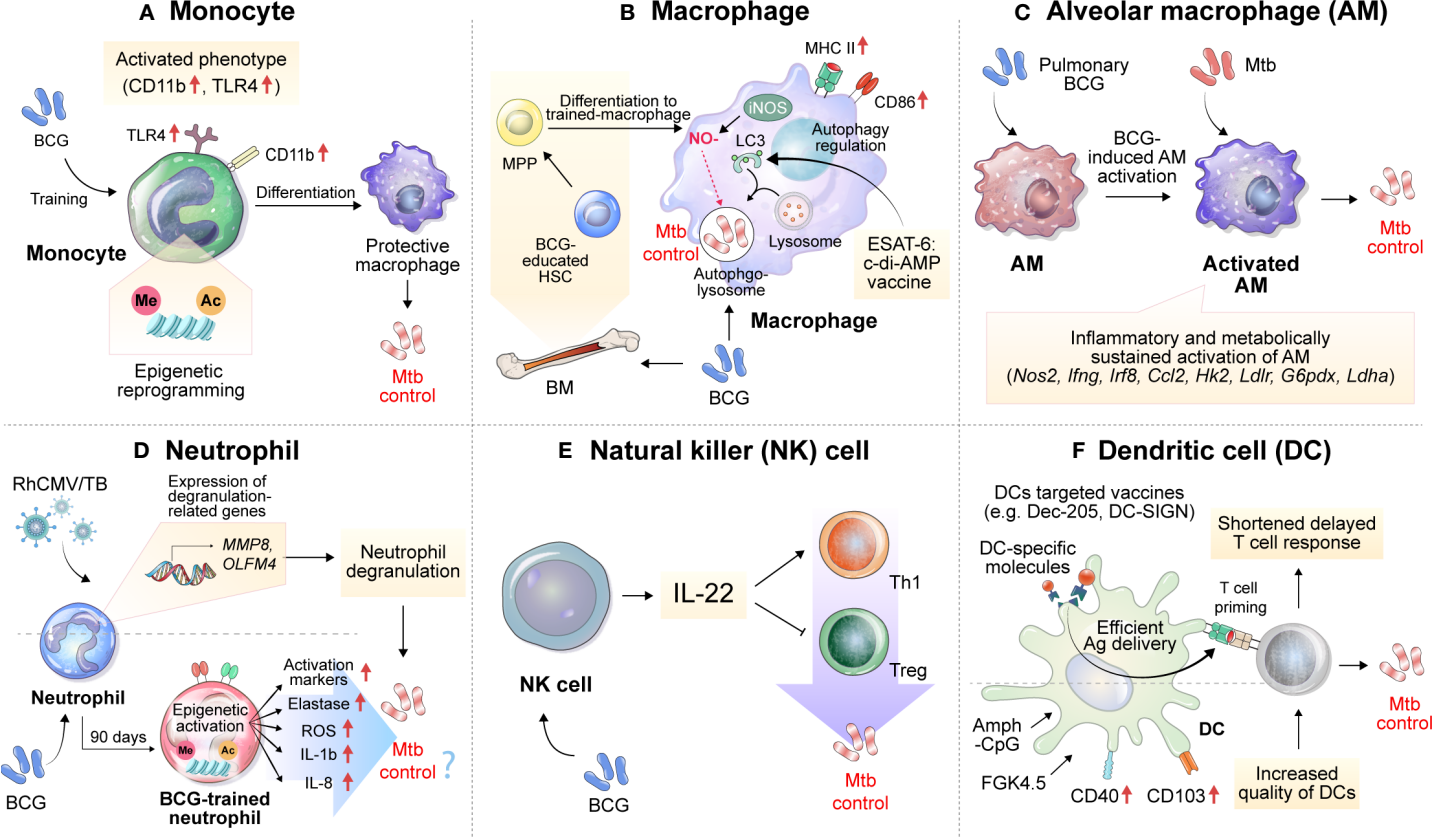
Implications of innate immune cells and relevant immune responses for the development of TB vaccines. Immune responses related to TB vaccines are mainly focused on adaptive immunity, especially T cells, but many studies imply the importance of innate immune responses. **(A)** BCG vaccination induces the histone epigenetic reprogramming of monocytes, resulting in an activated phenotype with increased CD11b and TLR4 expression. **(B)** BCG can activate macrophages and educate hematopoietic stem cells (HSCs) to differentiate into more protective macrophages against Mtb infection. Vaccination with ESAT6:c-di-AMP can control Mtb growth by regulating autophagy. **(C)** Alveolar macrophages, which act as first-line defenders against pathogens entering the lungs, are inflammatory and sustainably metabolically activated by BCG mucosal vaccination, which controls the dissemination and growth of Mtb. **(D)** The expression of genes related to neutrophil degranulation such as *MMP8* and *OLFM4* was suggested as a correlate of protection in the RhCMV/TB-vaccinated rhesus macaque model. BCG vaccination in healthy humans induces long-lasting changes in the neutrophil phenotype, characterized by increased expression of activation markers and antimicrobial function, which is associated with genome-wide epigenetic modifications in trimethylation at lysine 4 on histone 3. The enhanced function of human neutrophils persists for at least 3 months after vaccination. **(E)** Depletion of NK cells during BCG vaccination reduces protection against Mtb infection, concomitant with decreased Th1 response and increased Treg levels. The complementation of IL-22 restores the vaccine efficacy of BCG against Mtb infection, which was reduced by NK cell depletion. **(F)** Vaccines targeting DCs by using DC-specific molecules, such as Dec-205 and DC-SIGN can effectively deliver Ags to DCs. An increase in the quality of DCs through treatment with Amph-CpG and FGK4.5 can increase vaccine efficacy via effective T-cell priming. MPP, multipotent progenitor; HSCs, hematopoietic stem cells; NO, nitric oxide; iNOS, inducible nitric oxide synthase; c-di-AMP, c-di-adenosine monophosphate; BM, bone marrow; LC3, microtubule-associated protein 1A/1B-light chain 3; Amph-CpG, amphiphilic form of CpG.

Continuous research on innate immune factors related to the efficacy of TB vaccines has been conducted. Strategies that target these innate immune factors have been shown to improve vaccine efficacy. In addition, the characteristics of innate immune factors that correlate with vaccine efficacy show potential as biomarkers of vaccine efficacy (**Figure 1**).

#### 5.1.1 Monocytes

The monocytes to lymphocytes (ML) ratio in peripheral blood has been reported to be correlated with TB disease risk among HIV-infected patients (131, 132). In addition, it was reported that the ML ratio increased in severe TB patients and more so in males than females even within the TB patient group, showing a correlation with TB progression (133). Interestingly, Zelmer et al. reported that upon inoculating inbred mouse strains of different genetic backgrounds with BCG, the ML ratio correlated with BCG-induced vaccine efficacy against Mtb infection, suggesting that monocytes are deeply involved in the vaccine-induced immune response (31). BCG vaccination induces an increase in inflammatory mediator production by monocytes through histone modifications and specific gene activation (134). After BCG immunization, circulating monocytes in healthy volunteers released two times more cytokines, such as IL-1β and tumor necrosis factor (TNF)-α, upon stimulation with TB nonspecific pathogens. These BCG-trained monocytes had increased expression of CD11b and Toll-like receptor 4 (TLR4), and these immune effects were related to histone epigenetic reprogramming induced by activation of the NOD2 receptor to increase trimethylation of lysine 4 on histone 3 (H3K4m3) (**Figure 1A**). Interestingly, the effectiveness of trained immunity was maintained for up to one year, and heterogeneous protection by BCG vaccination in terms of neonatal death from other infectious diseases was significantly increased in the infant group aged 1 to 5 years (135). Recently, protection by BCG revaccination has been reported at the clinical stage (50), but the specific protective mechanism has not yet been fully elucidated.

#### 5.1.2 Macrophages

Recently, vaccination with ESAT6:cyclic diadenosine monophosphate (c-di-AMP) was shown to cause significant reductions in the bacterial burdens of the lungs and spleens in a mouse model by regulating autophagy in Mtb-infected macrophages (136). In addition, mouse bone marrow-derived macrophages infected with BCG become epigenetically modified to provide better protection against Mtb infection (137). This macrophage activation phenotype was also reported by Mate et al., and increases in MHC II, CD86, and inducible nitric oxide synthase levels were observed after intranasal (IN)-BCG vaccination but not after subcutaneous (SC) vaccination (**Figure 1B**).

Alveolar macrophages (AMs) may serve as the first line of defense against respiratory pathogens. However, a mouse model study showed that AM depletion has a protective effect on lung Mtb infection (138). Mtb becomes an exclusive niche for up to 10 days after Mtb infection (139). In addition, Mtb induces a Th1 response by inducing rapid dissemination of bacilli to the lymph nodes in an IL-1 receptor-dependent manner after AM infection, but poorly transmissible Mtb delays this process, residing inside AMs and developing and promoting the Th17 response (140). These reports suggest that AMs induce a delay in the early immune response during Mtb infection, leading to a delay in protective T-cell immunity.

On the other hand, it has been reported that mucosal vaccination with BCG is effective in inhibiting early dissemination of Mtb by inducing activation while BCG is present in AMs (141). In addition, the formation of trained immunity in mouse AMs through vaccination or infection has been reported (142, 143), and in this context, pulmonary BCG vaccination increases Mtb growth control in AMs early in infection and, through IL-1 signal-dependent Mtb transmission (140), may lead to shortening of the T-cell response delay (**Figure 1C**). Recently, aerosol vaccination with a human serotype-5 adenovirus (Ad)-vectored TB vaccine (AdHu5Ag85A) was reported in a clinical phase 1b trial. Transcriptomic analysis of AMs isolated from the aerosol AdHu5Ag85A-immunized group in this study showed that aerosol vaccination with AdHu5Ag85A induced persistent transcriptional changes in AMs related to the response to anoxia, inflammatory response to Ag stimulation, tyrosine phosphorylation of signal transducer and activator of transcription proteins, regulation of IL-10 production, response to IL-1 and histone demethylation (144).

#### 5.1.3 Neutrophils

The importance of neutrophil in TB is evidenced by the identification of prominent neutrophil transcription signatures in the blood of TB patients (145). The formation of neutrophil extracellular traps (NETs) induced by type I IFN promotes bacterial growth and disease severity in Mtb-infected mice (146). Given the critical function of neutrophils in TB pathogenesis, it is important to understand their properties in vaccine immune responses.

Monalisa et al. reported that the depletion of neutrophils during vaccination with *Mycobacterium smegmatis* expressing CMX induced a decrease in protection against Mtb infection in a mouse model, with a decrease in Th1 and Th17 responses in lung tissue and spleen, suggesting the function of neutrophils in the formation of T-cell responses (147). Thomas et al. reported the role of neutrophils in the formation of protective immunity by BCG vaccination (148). Seven days after BCG inoculation via the SC route, a slight increase in the frequency of neutrophils was observed in the lung tissue. In addition, the protective immunity induced by BCG was independent of T cells, and it was reported that this effect was maintained until 30 days after vaccination in T cell- or TNF-α-deficient mice. After BCG inoculation, depletion of neutrophils using an anti-Ly6G antibody resulted in protection provided by BCG being reduced by half, and this phenomenon was confirmed regardless of the presence of neutrophils at the time of Mtb infection (148). These results suggest that neutrophils contribute to the generation of protective innate immunity in the early stage of infection rather than direct killing of Mtb. In addition, BCG vaccination of healthy individuals generated phenotypic alterations in neutrophils, with enhanced antimicrobial function as well as upregulation of activation marker expression. The change in human neutrophils lasts for at least three months, along with genome-wide epigenetic remodeling via H3K4m3 modifications (149) (**Figure 1D**).

Recently, Hansen et al. administered the rhesus cytomegalovirus vectors (RhCMV) encoding Mtb Ag inserts (RhCMV/TB) vaccine to BCG-vaccinated or unvaccinated rhesus macaques (150). Before Mtb-challenge, the transcriptomic analysis of whole blood revealed that the gene expression levels predictive of the RhCMV/TB vaccine effect were predominantly from neutrophils. These genes were linked to innate immunity and pathways related to neutrophil degranulation, which encompassed genes encoding neutrophil granule effector molecules such as MMP8 and CTSG (**Figure 1D**). However, in the group vaccinated with BCG + RhCMV/TB, a specific set of genes associated with protection, such as *MMP8*, *CTSG*, and *CD52*, showed reduced expression compared to the group vaccinated with RhCMV/TB alone at the early phase of Mtb challenge. These transcriptional profiles correlated with a lower protective ability of BCG + RhCMV/TB than RhCMV/TB vaccine.

#### 5.1.4 Natural killer cells

NK cells accumulate in the lungs during Mtb infection and produce IFN-γ and perforin, but studies on the function of NK cells in vaccine responses are still lacking. BCG-vaccinated mice had an increased number of NK cells in the spleen and peripheral lymph nodes. To determine the function of BCG-induced NK cells, anti-NK1.1 antibodies were administered to BCG-vaccinated mice to deplete NK cells, resulting in decreased protective efficacy of BCG and an increased number of regulatory T cells (Tregs) and a diminished T-cell response (151). The depletion of NK cells resulted in the induction of Tregs and a reduction in T-cell activity after Mtb infection, but supplementation with recombinant IL-22 rescued BCG-induced protection, suggesting the importance of IL-22 in NK cell-mediated protection against BCG vaccination (151) (**Figure 1E**). On the other hand, Thomas et al. infected mice with H37Rv after depleting NK cells by treatment with an anti-asialo-GM1 antibody during BCG vaccination but found no difference in efficacy after 30 days (148).

#### 5.1.5 Dendritic cells

Delayed T-cell responses are one of the typical characteristics of TB, and to control them, the formation of a protective T-cell response and the accumulation of a considerable number of T cells at the site of inflammation are important. In this process, proper dendritic cell (DC) activation, rapid DC migration, and interaction with T cells are important. According to previous studies with a mouse model, vaccination relieved the delayed T-cell response of the host to some extent, but a delayed CD4^+^ T-cell response still occurred in the vaccinated host (152, 153), which may be the reason why vaccine-induced TB control is not effective. There have been studies that have focused on the role of DC frequency or activation in the delay of T cell response in vaccination. In a mouse model, vaccination with recombinant BCG-producing FMS-like tyrosine kinase 3 ligand or granulocyte-macrophage colony-stimulating factor (GM-CSF) increased the frequency of DCs. This increase in DC frequency demonstrated enhanced protection against Mtb infection (154, 155). In addition, in the analysis of the RNA expression profile related to vaccine immunogenicity and efficacy in the PBMCs of recipients of the TB vaccine candidate M72/AS01_E_, it was confirmed that the increase in the number of activated DCs was induced by vaccination (156). Griffiths et al. reported that after BCG vaccination, an increase in CD103 and CD40 expression on DCs induced through CpG and anti-CD40 antibody (FGK4.5) stimulation increased the number of DCs and strengthened the interaction ability with T cells in the lung, resulting in increased vaccine efficacy against Mtb infection (157) (**Figure 1F**). These findings indicate that increasing the frequency or quality of DCs can directly affect vaccine efficacy. Moreover, efficient Ag delivery is also directly related to the efficacy of the TB vaccine. Griffiths et al. confirmed that the transfer of DCs loaded with Mtb Ag85B accelerated the delayed T-cell response of mice immunized with BCG or Mtb Ag and increased the vaccine efficacy, showing the importance of DCs in vaccination (157). DC-targeted vaccines through DC-specific molecules such as DEC-205 or DC-SIGN (158, 159) show increased T cell response and vaccine efficacy in mouse model, which emphasis the importance of DCs in TB vaccination (**Figure 1F**). However, there are still few data on the response of DCs induced by vaccines.

### 5.2 Correlates of protection in adaptive immune response to the TB vaccine

Protection against Mtb afforded by a TB vaccine in a mouse model appears to correlate with the T_CM_ phenotype, but data are limited. Tissue-resident memory T (T_RM_) cells, parenchymal-resident noncirculating memory cells that have been studied only relatively recently, reside in tissues for early recognition of infected cells (160). Vaccines that elicit a rapidly accessible T-cell response to the pathogen early in Mtb infection are thought to enable more efficient protection via T_RM_ or T_EM_ cells. Furthermore, the protective role of antibodies in the pathogenesis of TB highlights the need for continuous exploration of the adaptive immune response as a biomarker for vaccine efficacy.

#### 5.2.1 Tissue-resident memory T cells

**Figure 2:**
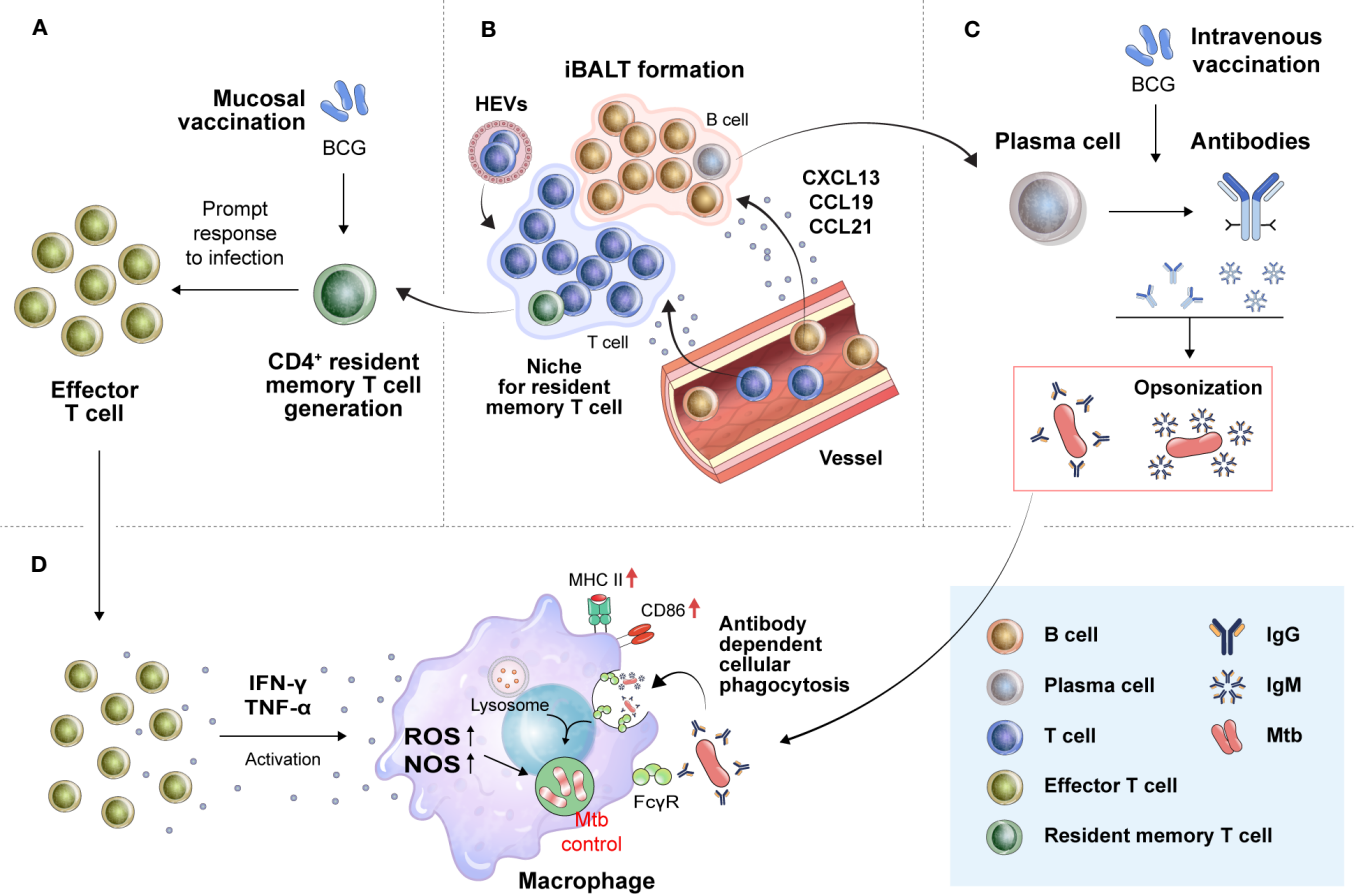
Translation of novel immune correlates found in preclinical animal models into humans for the development of more effective TB vaccines. **(A)** Mucosal vaccination can strongly induce T_RM_ cell development. In the early stage of Mtb infection, T_RM_ cells proliferate promptly to effector T cells to combat the bacilli by secreting proinflammatory cytokines. **(B)** T_RM_ cells residing in the lung parenchyma, especially CD4^+^T_RM_ cells, are known to be located in tertiary lymphoid structures, such as iBALTs. The formation of iBALTs is regulated by cytokines (IL-17, IL-22, and IL-23) and chemokines (CCL19, CCL21, CXCL12, and CXCL13). Structured iBALT consists of the T-cell zone and B-cell zone. These ectopic lymphoid-like structures provide a place where follicular helper T cells mediate the selection and survival of B cells, resulting in the differentiation of long-lived plasma cells. These processes make it possible to induce *in situ* protective humoral immunity by secreting protective immunoglobulins, such as IgM and IgG. **(C)** These humoral and cellular *in situ* immune responses can create an environment favorable for the host to control Mtb. Immunoglobulins from B cells aggregate Mtb, resulting in the formation of pathogen-antibody complexes. **(D)** The formation of these complexes enhances phagocytic killing activity of macrophages. Proinflammatory cytokines from T cells (IFN-γ and TNF-α) activate macrophages to kill Mtb. T_RM_ cells, tissue-resident memory T cells; iBALTs, inducible bronchus-associated lymphoid tissues; ROS, reactive oxygen species; NOS, nitric oxide synthase; FcgR, Fc gamma receptor.

Since Mtb is transmitted via the aerosol route, generating a pulmonary memory immune response is important for protective immunity, which enables an immediate immune cell response to an infection site. The generation of T_RM_ cells has been shown to correlate with protection against Mtb and is characteristically induced, particularly upon mucosal vaccination (161, 162) (**Figure 2A**). Mucosal transfer of T_RM_ cells from BCG-vaccinated mice to naïve mice showed that both CD4^+^ and CD8^+^ T_RM_ subpopulations can provide partial protection against Mtb infection (163) and can restrict intracellular Mtb survival *in vitro* (164). A recent study using human tissue from surgically resected lungs also demonstrated that the number of IL-17-producing Mtb-specific T_RM_-like cells in the lungs was inversely correlated with IL-1β levels in the blood, indicating that Mtb-specific T_RM_ cells producing IL-17 may play an important role in controlling Mtb in the human lung (165). These reports suggest that T_RM_ cells are correlated with protection in TB vaccination models, and this cell population could be a target for vaccine strategies for protection against TB. In several disease models, including TB, a strategy called “prime and pull” to recruit memory T cells through booster vaccination to target tissues after prime vaccination has been carried out (166–168). Roces et al. reported a vaccine strategy to boost immunization with H56 protein in the lung mucosa through inhalation after immunization with CAF01:H56, a clinical TB vaccine candidate, by the SC route (169). For the booster vaccination, the poly lactic-co-glycolic acid delivery system, which has been recognized for its safety, was used, and the experiment was designed based on the manufacturing of a powder containing H56. Haddadi et al. parenterally immunized mice using a recombinant replication-deficient chimpanzee Ad-based TB vaccine expressing Ag85A (AdCh68Ag85A) and then immunized the mice with Ag85 complex via the IN route (170). In this study, immunization with Ag85 via the IN route was able to induce an almost 2-log reduction in the bacterial burden in the lung tissue upon Mtb H37Rv infection compared to that in the group that received only parenteral immunization. Importantly, these results demonstrate that the prime and pull strategy for the respiratory mucosa can promote the development of T_RM_ cells as well as the recruitment of Ag-specific T cells into lung tissue. In addition, to effectively pull memory T cells into the respiratory mucosa, it was confirmed that booster vaccination should be given at a time when T cells mainly form a memory type rather than after a short period of time when effector T cells are mainly present after prime vaccination (170).

Direct immunization to the lung, the site of infection, has been reported to be beneficial for the formation of T_RM_ cells. However, Darrah et al. confirmed the level of T_RM_ cells in lung tissue 4 weeks after BCG immunization in an NHP model, BCG delivered by the intravenous (IV) route was able to induce higher levels of T_RM_ cells than BCG injected by the aerosol route (171). Of note, six months after Mtb challenge, nine out of ten macaques with BCG immunization via the IV route produced a significant Ag-specific T-cell response accompanied by highly protective vaccine efficacy compared with those with the intradermal (ID) or aerosol route vaccination; and six of ten macaques administered BCG via the IV route had no detectable levels of infection (171).

The formation of T_RM_ cells was thought to proceed via differentiation from effector T cells *in situ* via transforming growth factor beta (TGF-β) and IL-15 signals when inflammation resolves (172). In addition, at the priming level, mouse Batf3-dependent DCs and human CD1c^+^CD163^+^ DCs producing TGF-β can prime T cells for T_RM_ cell generation in lymphoid tissues (173). These reports may reveal the reason for the finding that systemic immunization via the IV route induces higher T_RM_ cell levels in lung tissue than the aerosol route or direct BCG delivery to the lungs. However, it is a challenge to ensure the safety of the administration method, to analyze the T_RM_ cells and to verify the efficacy in clinical trials.

The generation of inducible bronchus-associated lymphoid tissues (iBALTs), a type of tertiary lymphoid structure (TLS), could be pivotal because increased CXCR5^+^ CD4^+^ T cell levels were correlated with a better outcome of TB disease (174, 175) (**Figure 2B**). TLSs are formed at sites of infection or chronic inflammation and have also been found in autoimmune disease, allograft rejection, and cancer. Importantly, B cells that respond to tumor-associated TLSs appear to participate in antitumor immunity, as B cells cultured from TLS-containing biopsy samples produced tumor Ag-specific antibodies (176). This ectopic lymphoid structure can also act as a site for B-cell selection and maturation (177) and can provide a niche for memory B cells and T cells (178). In an influenza virus-infected mouse model, CD8^+^ T_RM_ cells were mainly located in the interstitium extending into the lung parenchyma, whereas CD4^+^ T_RM_ cells were found in the iBALT niche (179, 180). These reports suggest that iBALT might enable a rapid and effective response to Mtb infection.

#### 5.2.2 Effector memory T cells

Hansen et al. recently tested RhCMV/TB vaccine capable of expressing six or nine Mtb Ags in rhesus macaques (150). Upon infection of the macaques with the Erdman strain almost 1 year after vaccination, it was confirmed that sterile immunity was induced in approximately 40% of the experimental group animals (150). In contrast to previous vaccine strategies aimed at eliciting primarily T_CM_ cell responses, this CMV-based vaccine elicited primarily T_EM_ cell responses. T_EM_ cell population appears to be maintained by continuous restimulation of Mtb-specific T cells by periodic reactivation of the cytomegalovirus, and the authors suggest that protective immunity induced by the RhCMV/TB vaccine can be induced by Mtb-specific T cells from the vaccination. This phenomenon is thought to be due to the high frequency of T_EM_ cells generated by the restimulation of cells (150). However, CMV infection can be fatal in immunocompromised humans (181), and according to a recent study conducted in South Africa, CMV seems to be related to the increase in the incidence of TB in children (182). It is believed that replicating CMV-based vaccines will be needed, but this type of vaccine needs to be proven effective.

#### 5.2.3 IgG/IgM

While the role of humoral immunity in TB has been controversial, several reports have led to a reassessment of the significance of antibody-mediated immunity in providing protection against Mtb (183–185). Antibodies can play an important role in preventing or eliminating the initial Mtb infection (**Figure 2C**). Antibodies can bind Mtb and increase macrophage phagocytosis by binding Fc receptors and play an effective role in clearing other intracellular pathogens. Recent studies have shown that antibodies that prevent Mtb infection are present in humans (183, 185). The first suggestion that antibodies may be protective was reported by Teitelbaum et al. (186). Mtb was pretreated with two monoclonal antibodies specific to cell surface Ags and injected through the trachea. Pretreated bacterium-infected mice lived substantially longer than control mice (186). Moreover, Ag85A-specific IgG responses have been associated with reduced TB development (187). Alternative vaccination routes, such as mucosal and IV, result in the production of pulmonary IgA and iBALTs, reducing the bacterial burden (161, 162, 171). Recently, Edward et al. reported that IgM has a negative correlation with Mtb load upon IV-BCG vaccination in macaques (188). They examined antibody responses across several BCG vaccine regimens in NHP models to determine if particular antibody profiles were linked with better Mtb control. Correlation analysis revealed a particularly strong association between plasma and bronchoalveolar lavage IgM responses and reduced Mtb burden upon BCG vaccination. Importantly, elevated Ag-specific IgM titers were observed not only in the lungs but also in the plasma of the IV-vaccinated animals. Furthermore, IgM antibodies enhance the Mtb restriction activity *in vitro*. These reports show the potential of IgG or IgM as a new COP in clinical practice.

These humoral and cellular *in situ* immune responses can be targeted for induction by a TB vaccine. Such a strategy can create a favorable environment for the host to control Mtb. Pathogen-antibody complexes formed by IgM or IgG secreted by B cells can promote the phagocytosis of Mtb by macrophages. A rapid and appropriate T-cell response to infection can induce Mtb control by inducing the activation of macrophages in the early stages of infection (**Figure 2D**).

### 5.3 Key compensatory markers

#### 5.3.1 Biomarkers in urine samples

Finding a biomarker for COPs of a TB vaccine in urine offers several advantages over blood with respect to collection and safety. In particular, the development of biomarkers for COPs through urine sampling can be helpful in controlling TB by using vaccines in the least-developed countries, especially in the least-developed countries with a high incidence of TB, because vaccination subjects can continually collect samples themselves after simple education.

The possibility of identifying COPs for TB vaccination via biomarkers in urine is suggested by urine analysis studies on indicators of TB development and treatment. For example, in active TB patients, inflammatory mediators such as IL-8, IL-2, TNF-α, IFN-γ, chemokine ligand (CCL) 5, macrophage inflammatory protein-1 alpha and beta were not detected in the urine, but chemokine (C-X-C motif) ligand (CXCL) 10 was persistently detected (189). Moreover, the level of CXCL10 in urine was decreased in patients treated with TB drugs compared to that in active TB patients (189, 190). However, the CXCL10 level in urine is not a specific biomarker that is increased only by pulmonary TB infection, but it can be used as a limited biomarker because it shows a similar increase in patients with other lung diseases. Lipoarabinomannan (LAM), an Mtb cell wall component detected in urine, was used to establish a urinalysis for diagnosing disseminated TB patients among human immunodeficiency virus (HIV)-infected patients (191, 192). In addition, changes in the levels of 12 metabolites in urine were reported in patients with active TB after anti-TB treatment (193).

Biomarker analysis using urine samples is also being applied in vaccine research. To evaluate the toxicity of two influenza vaccines with different toxicities in a mouse model, hydrogen-1 nuclear magnetic resonance spectroscopy was used to observe changes in urine metabolite levels, and findings were compared with existing toxicity indicators such as weight loss and leukopenia (194). In addition, analysis of changes in urinary cytokine levels, as a predictor of immunogenicity and reactogenicity, induced by the AS01_E_-adjuvanted hepatitis B vaccine in healthy adults was used to evaluate the effectiveness of the vaccine (NCT01777295). Upon measuring the concentrations of 24 cytokines in the urine of the saline-administered control group and the vaccine group, a transient increase in CCL2 and CXCL10 levels was observed after vaccination (195). These results show the possibility of discovering biomarkers as COPs for TB vaccination through urine analysis.

#### 5.3.2 Type I IFNs

Detrimental roles of type I IFNs in TB pathogenesis have been extensively investigated. However, there have also been reports on the protective role of type I IFNs in relation to TB vaccines. For example, it has been reported that type I IFNs can increase the immunogenicity of the BCG vaccine in mouse models. These reports showed that vaccination with ESX-1-expressing BCG could increase vaccine efficacy against Mtb infection in murine models by increasing ESX-1-dependent type I IFN production (196, 197). In the case of MTBVAC, the double deletion of *phoP* and *fadD26* resulted in a 25- to 45-fold increase in c-di-AMP levels compared to those with Mtb or BCG, which resulted in attenuation of toxicity and high vaccine efficacy in a mouse model through an ESX-1 system-dependent type I IFN response (198, 199). Additionally, vaccination with BCGΔBCG1419c in mouse models had a higher vaccination efficacy than normal BCG vaccination (200). The BCG1419c gene encodes a cyclic diguanosine monophosphate (c-di-GMP) phosphodiesterase that normally functions to hydrolyze c-di-GMP. Vaccination with BCGΔBCG1419c is expected to result in the production of more c-di-GMP, which is thought to have a protective effect by inducing an increase in type I IFN signaling through the TANK-binding kinase 1 and interferon regulatory factor 3 cascade (200, 201). In addition, the administration of IFN-α in combination with BCG vaccination has been shown to increase the efficacy of the TB vaccine in a mouse model (202). These results indicate that type I IFNs may have different functions in TB pathogenesis and vaccine-induced immunity and the positive role of type I IFNs shows their potential as biomarkers for the efficacy of vaccine candidates.

## 6 The fifth hurdle: further considerations

### 6.1 Factors affecting vaccine efficacy: preexposure to related and unrelated pathogens

#### 6.1.1 Helminth

Chronic infection with helminths has been well documented in cells secreting IL-10 or TGF-β and induces the induction of Tregs, which downregulate both Th1 and Th2 immune responses and mainly interfere with the function of effector Th1 cells (203). These immunological properties of helminths can affect the efficacy of TB vaccines. BCG immunogenicity was found to be lower in individuals with helminth infection than in those treated with anti-helminthic drugs (204). The reduced responses were associated with decreased purified protein derivative (PPD)-specific IFN-γ and IL-12 production and with an enhanced PPD-specific TGF-β response rather than an increase in the PPD-specific Th2 response itself. Likewise, helminth-infected college students aged 18 to 24 years in Ethiopia who received deworming therapy prior to BCG vaccination displayed relatively more PPD-specific immune responses than untreated control individuals (205). Similarly, maternal infection with helminths during pregnancy negatively influenced the frequency of IFN-γ-producing T cells in the cord blood of neonates (206) as well as the development of Th1 immunity in offspring vaccinated with BCG (207). Recently, Schick et al. reported that *Nippostrongylus brasiliensis* infection-induced production of IL-4 or IL-13 suppressed the H1/CAF01 vaccination-induced Th1/Th17 response in a mouse model (208). These reports show that immunization by vaccination or infection with helminth after vaccination can affect vaccine efficacy. This problem is prominent in most of the world’s tropical and subtropical developing countries that have populations that are susceptible to helminth infection.

#### 6.1.2 Nontuberculous mycobacteria

**(NTM):** NTM have been reported to have cross-reactivity with BCG in humans (209), which is thought to be a factor that may affect the vaccine efficacy of BCG. BCG vaccination has been reported to show some protective effects against NTM infection in humans (210). In a mouse model, exposure to NTM after BCG vaccination also enhances BCG efficacy against Mtb infection (211), suggesting that the impact of NTM infection on BCG efficacy varies depending on factors such as the timing of exposure, route of infection, and viability of NTM. However, prior sensitization to NTM has the potential to stop BCG proliferation, prevent the induction of an effective BCG-directed immune response, and ultimately inhibit the protective effect against Mtb infection (212). Another study reported that oral exposure to *Mycobacterium avium* after BCG vaccination reduced the efficacy of BCG vaccination against Mtb infection in a mouse model (213). Humans are inevitably exposed to NTM via multiple infection sources such as shower water, soil, and pool water. Thus, the effect of NTM exposure and its precise mechanism of action on immunological responses are worth further investigation.

### 6.2 Vaccine application in the context of underlying diseases

TB is the leading cause of death among people living with HIV, affecting the immune system and eventually waning defense systems against infections, leading to an increase in the risk of TB. It is well-reported that people living with HIV are more than 20 times more susceptible to developing active TB. Therefore, protection against these two diseases is of complementary importance. Because of HIV-related immunosuppression, the TB vaccine may be less immunogenic and less effective in people with HIV infection than in people without HIV infection (214). Because HIV-infected people are a large subpopulation at a high risk of TB infection and disease, it is important to include them in TB vaccine trials. A vaccine that is expected to have a protective effect against HIV is being developed based on a promising vaccine candidate in the TB vaccine clinical stage or BCG vaccine (215).

Diabetes prevalence affects TB incidence and TB mortality, resulting in two to three times the probability of developing TB, two times the risk of death during TB treatment, four times the risk of TB recurrence after completion of treatment, and two times the risk of infection with multidrug-resistant TB (MDR-TB). A cohort study reported that the longer the period of diabetes was, the more associated with TB disease, and TB was more commonly identified in patients with a fasting plasma glucose level over 202 mg/dL (216). In addition, it was confirmed that the higher the glucose concentration in the blood of diabetic patients was, the weaker the adaptive immune response to Mtb (217). Verma et al. established a latent TB infection mouse model and induced diabetes in Mtb-infected mice by administering streptozotocin to investigate the relationship between latent TB and diabetes. These hyperglycemic conditions led to a decrease in MCP-1 and MMP9 levels and increased MMP1 levels in latent TB infection, which may lead to reactivation of latent TB infection by disrupting granulomas (218). Clement et al. reported that metabolic stress caused by hyperglycemia decreases Ag presentation ability and inhibits the proliferation of CD4^+^ T cells (219). These reports suggest the possibility that diabetes can affect the formation of TB vaccine-induced protective immunity.

### 6.3 Vaccination for elderly people

In old age, lung structural degeneration as well as changes in immune cell functions make people vulnerable to respiratory diseases, and these age-related immunological changes may also affect vaccine efficacy. The incidence of TB is common in elderly individuals and increases progressively with age, and mortality from TB is also higher in older patients (220). This phenomenon is related to the reactivation of lesions from a dormant state, which is affected by changes in the immune system with aging. In addition, chronic inflammation in aging individuals disrupts T-cell responses, followed by decreased vaccine efficacy. For example, the application of a delayed-type hypersensitivity model of BCG vaccination and TST of aged NHPs showed that the immune response to antigenic challenges between the tissue site and the periphery is compromised, restricting the optimal immune memory response (221). A follow-up study showed reduced or delayed T-cell recall responses to lung infection sites in aged BCG-vaccinated rhesus macaques (222).

Recently, nonspecific protective efficacy of the BCG vaccine was confirmed against respiratory diseases such as COVID-19 through immunological changes favorable to respiratory infections in elderly people (223, 224). Many findings reveal that this nonspecific protection is generated from innate immune memory via metabolomic and epigenetic reprogramming, also known as trained immunity. Blood samples before and 1 month after BCG vaccination were compared in 82 subjects between the ages of 60 and 80 years (225). It was confirmed that BCG vaccination induced reductions in the levels of pro-inflammatory cytokines (TNF-α, IL-6, IL-1β) and chemokines (CCL2 and CXCL10), acute phase proteins such as C-reactive protein, and matrix metalloproteinases (225, 226). Considering the immune activation by BCG vaccination in elderly people and the positive results of BCG revaccination, BCG revaccination in elderly people may be a beneficial strategy to reduce elderly mortality due to TB.

### 6.4 Oral vaccination: an alternative route for TB vaccine

TB vaccine candidates currently in the clinical stage are vaccinated through the intramuscular (IM) route or ID route. In addition, studies with noteworthy results in preclinical stages through the aerosol route or the intravenous route have recently been reported. However, studies on oral route vaccination are still limited. In the case of BCG, since the safety of the ID route of BCG for mass vaccination was confirmed by Scandinavian researchers in the 1930s, it has been used until now (227). However, the BCG vaccine was initially developed as an oral vaccine and was used in that form until an incident in Germany in 1930, when the oral BCG vaccine was contaminated with Mtb. In Brazil, oral BCG vaccination was administered to newborns until 1976. Recently, Hoft et al. demonstrated the safety of oral BCG vaccination through a comparative analysis of 68 healthy adults who received BCG via the intradermal (ID) route and the oral route (228). ID-BCG vaccination induced a higher systemic Th1 response than the oral route. In contrast, oral route BCG vaccination produced more elevated Mtb-specific secretory IgA and Mtb-specific bronchoalveolar lavage T cell responses than ID-BCG vaccination (228). A lipid-based formulation has been developed for oral BCG vaccination, and the results of vaccination in BALB/c mice showed increased vaccine efficacy compared to conventional BCG vaccination (229). In addition, to increase the efficacy of oral vaccination of lipid-formulated BCG vaccination, improved vaccine efficacy was confirmed through aerosol infection after oral vaccination with recombinant BCG expressing Ag85B-ESAT6 fusion protein in a guinea pig model (230). The effectiveness of oral vaccination was also confirmed with a subunit vaccine model. Although oral immunization was less effective as a priming vaccination of fusion protein ESAT-6-Ag85B with detoxified monophosphoryl lipid A (MPL), heterogeneous priming and boosting vaccination strategies combined with oral boost induced significant systemic Th1 response, providing protection similar to or exceeding vaccination via the SC route against Mtb infection (231).

Oral vaccination is an appealing route due to the absence of needles, which eliminates the risk of cross-infection, and the ability to administer vaccines without the need for specialized healthcare professionals. Exploring the properties of this unconventional vaccine route presents an additional potential strategy for TB vaccination.

## 7 Conclusions

Despite recent progress in clinical trials of several vaccine candidates and anti-TB drugs, the World Health Organization (WHO)’s “End TB strategy” milestone of the year 2025 has become challenging due to the coronavirus disease 2019 (COVID-19) pandemic. With the continued high prevalence and the death rate returning to the levels observed 10 years ago, researchers’ endeavors to find novel strategies to combat TB have been crippled. Nevertheless, advanced knowledge on new immune factors and consistent efforts to develop vaccine candidates will reveal promising ways to combat TB.

Most vaccine studies have focused on Th1 cells and the effector cytokine IFN-γ as potential indicators of vaccination success and vaccine efficacy. However, as the protective functions of IL-10, which have been considered negative, or novel protective functions of Th17 cells have been revealed through numerous studies, the narrow view of vaccine immunity has been expanded. From this point of view, the understanding of the functions of currently known factors is unlikely to be complete, as the factors can perform different functions in a temporally and spatially diverse immune environment. Novel analyses, such as those based on novel immune indicators, metabolomics and transcriptome analysis, may provide further insight into the complex immune environment and control of TB with vaccines. Furthermore, to progress beyond the existing ‘one-size-fits-all’ treatment approach, the prescription of a treatment strategy classified according to the patient’s condition is being considered (232, 233). These considerations should account for individual characteristics such as underlying disease and epidemiological status. For example, live attenuated vaccines, including the BCG vaccine, can be lethal in HIV-positive patients. In particular, elderly people over the age of 65 years who are very vulnerable to infection can be an important target.

The seriousness of the recent COVID-19 crisis and the quick response of humans to overcome it provide a positive message for overcoming existing diseases such as TB. However, reports of side effects such as myocarditis and severe allergic reactions (234, 235) indicate that immune balance is an important consideration for vaccine development. More than 100 years have passed since Koch first identified Mtb, and many researchers have made efforts, with many advances, to control these vicious bacilli that have killed a tremendous number of people. With numerous vaccine candidates being evaluated in clinical trials, the direction of TB vaccine development seems much more sophisticated than in the past, but achieving the intended goal remains challenging. Although several candidates showing protective efficacy in animal models eventually failed to exhibit vaccine efficacy in clinical trials, the collection and analysis of data for each candidate, whether successful or not, are obviously valuable to reduce the probability of failure. In addition, the use of BCG or BCG revaccination should be maximized and optimized in combination with other types of vaccine candidates (236). Finally, heterogeneous vaccine strategies with candidates in different phases of clinical trials, such as adjuvanted subunit priming with a vector-based candidate boost, can be another strategy for better inducing pleiotropic protective immunity.

## Author contributions

HK and SS elaborated on the subject of the review. HK, H-GC, and SS wrote the manuscript. SS helped write the manuscript, provided helpful ideas and corrected the manuscript. All authors contributed to the article and approved the submitted version.
